# Transcriptome analysis identifies strong candidate genes for ginsenoside biosynthesis and reveals its underlying molecular mechanism in *Panax ginseng* C.A. Meyer

**DOI:** 10.1038/s41598-018-36349-5

**Published:** 2019-01-24

**Authors:** Mingzhu Zhao, Yanping Lin, Yanfang Wang, Xiangyu Li, Yilai Han, Kangyu Wang, Chunyu Sun, Yi Wang, Meiping Zhang

**Affiliations:** 1 College of Life Science, Jilin Agricultural University, 2888 Xincheng Street, Changchun, Jilin, 130118 China; 2 Research Center of Ginseng Genetic Resources Development and Utilization, 2888 Xincheng Street, Changchun, Jilin, 130118 China; 3 College of Chinese Medicinal Materials, Jilin Agricultural University, 2888 Xincheng Street, Changchun, Jilin, 130118 China

## Abstract

Ginseng, *Panax ginseng* C.A. Meyer, is one of the most important medicinal herbs for human health and medicine in which ginsenosides are known to play critical roles. The genes from the cytochrome P450 (CYP) gene superfamily have been shown to play important roles in ginsenoside biosynthesis. Here we report genome-wide identification of the candidate *PgCYP* genes for ginsenoside biosynthesis, development of functional SNP markers for its manipulation and systems analysis of its underlying molecular mechanism. Correlation analysis identified 100 *PgCYP* genes, including all three published ginsenoside biosynthesis *PgCYP* genes, whose expressions were significantly correlated with the ginsenoside contents. Mutation association analysis identified that six of these 100 *PgCYP* genes contained SNPs/InDels that were significantly associated with ginsenosides biosynthesis (*P* ≤ 1.0e-04). These six *PgCYP* genes, along with all ten published ginsenoside biosynthesis genes from the *PgCYP* and other gene families, formed a strong co-expression network, even though they varied greatly in spatio-temporal expressions. Therefore, this study has identified six new ginsenoside biosynthesis candidate genes, provided a genome-wide insight into how they are involved in ginsenoside biosynthesis and developed a set of functional SNP markers useful for enhanced ginsenoside biosynthesis research and breeding in ginseng and related species.

## Introduction

Ginseng (*Panax ginseng* C.A. Meyer) is one of the most important medicinal herbs for human health and medicine. Ginseng has been shown to have several bioactive components, including triterpene saponins, flavonoids, fructoligosaccharides and polysaccharides^1^, but the most valuable is ginsenosides, a diverse group of triterpene saponins that are produced in all tissues of ginseng^2^. Studies have shown that ginsenosides provide several benefits for human health, including immunomodulation^3^, anti-inflammation^4^, anti-tumor^5,6^, anti-hyperlipidemia^7^ and anti-amnestic and anti-aging activities^8^.

Therefore, isolation and characterization of the genes involved in the ginsenoside biosynthesis have been a major focus of ginseng research and breeding. However, only a few genes involved in this process have been thus far cloned, no molecular tools have been developed to manipulate the process for enhanced research and breeding, and little is known about the molecular mechanism underlying ginsenoside biosynthesis. The genes by far cloned that are involved in ginsenoside biosynthesis include those encoding squalene synthase (*SS*)^9^, cycloartenol synthase (*CAS*)^10^, squalene epoxidase (*SE*)^11^, dammarenediol synthase (*DS*)^12,13^, beta-amyrin synthase (*β-AS*)^14^, farnesyl phosphate synthase (*FPS*)^15^, UDP-glycosyltransferase (*UGT*)^16^, and cytochrome P450 (*CYP716A53v2*, *CYP716A52v2* and *CYP716A47*)^17–19^. These studies indicate that it is likely that additional genes, including those of the *CYP* gene superfamily encoding cytochrome P450, are involved in ginsenoside biosynthesis.

The cytochrome P450, *CYP450*, gene superfamily is known to be widely present in all organisms, including plants, animals and microbes^20^. They encode monooxygenases involved in endogenous oxidation reaction, such as lipids, steroidal hormones and cholesterol as well as in the degradation of xenobiotics such as insecticide and herbicide^21–25^. The *CYP450* genes also extensively participate in the modification of various secondary metabolites that potentially have pharmacological properties as well, endowing medicinal herbs with potential medical and economical values^26^. Over the last decades, the functions of the *CYP450* genes have been studied in the biosynthesis of various herbal components, such as ginsenosides^17–19^, artemisinin^27,28^, nootkatone^29^, olean-12-ene-3β-24-diol soyasapogenol B^30^, oleanolic acid^31^, flavonones^32^ and hyoscyamine^33^.

In this study, we identified 100 *CYP450* genes from ginseng, defined *PgCYP* genes, whose expressions were significantly correlated with variation of nine mono- and total-ginsenoside contents. We analyzed these 100 *PgCYP* genes by association study and identified five SNPs and three InDels from 6 of them that were significantly associated with the ginsenoside contents in the four-year-old roots of 42 genotypes. Finally, we characterized these six *PgCYP* genes in several aspects and examined their relationships with those published ginsenoside biosynthesis genes that were previously cloned from the *PgCYP* gene superfamily, *CYP716A53v2*, *CYP716A52v2* and *CYP716A47*^17–19^, and from other gene families, including *SS*^9^, *CAS*^10^, *SE*^11^, *DS*^12,13^, *β-AS*^14^, *FPS*^15^ and *UGT*^16^. Therefore, this study has identified six new candidate *PgCYP* genes involved in ginsenoside biosynthesis and developed functional SNP/InDel markers for these genes, which are important for advanced ginsenoside biosynthesis research and enhanced breeding in ginseng and related species.

## Results

### Identification of the candidate *PgCYP* genes involved in ginsenoside biosynthesis

Han *et al*.^17–19^ cloned three *CYP* genes from *P*. *ginseng*, defined *CYP716A53v2*, *CYP716A52v2* and *CYP716A47*, and showed that they were involved in ginsenoside biosynthesis by gene expression repression analysis using RNA interference (RNAi) technology. These results indicated that the *PgCYP* gene superfamily plays important roles in ginsenoside biosynthesis. Moreover, Syed *et al*.^34^ documented that most of the genes contained in a genome are subjected to RNA alternative splicing that often results in multiple transcripts from a single gene. These multiple transcripts are translated into different proteins having different biological functions. Therefore, we performed correlation analysis of the expression activities of all 440 *PgCYP* transcripts derived from 414 *PgCYP* genes that we previously identified^35^ with the variations of nine mono- and total-ginsenoside contents in the four-year-old roots of 42 cultivars to examine whether any of them is likely also involved in ginsenoside biosynthesis, in addition to *CYP716A53v2*, *CYP716A52v2* and *CYP716A47*^17–19^.

We hypothesized if a gene is involved in ginsenoside biosynthesis, its expression activity should be correlated or associated with ginsenoside contents in ginseng. To test this hypothesis, we first conducted correlation analysis of the genes previously cloned from ginseng that were shown to be involved in the ginsenoside biosynthesis between their expressions and the ginsenoside contents in the four-year-old roots of 42 ginseng cultivars. These previously cloned ginsenoside biosynthesis genes included *SS*, *CAS*, *SE*, *DS*, *β-AS*, *FPS*, *UGT* and three *CYP* genes (*CYP716A53v2*, *CYP716A52v2* and *CYP716A47*) (Supplementary Table S1). We extracted the expressions of every transcript of these ginsenoside biosynthesis genes from the root transcriptome database of the 42 ginseng cultivars (Supplementary Table S2)^36^ and conducted correlation analysis with the nine mono- and total-ginsenoside contents (Supplementary Table S3). The result showed that the expressions of 1–4 transcripts of each of the ten published ginsenoside biosynthesis genes were correlated with the variations of one or more of the mono- and total-ginsenoside contents at a significance level of *P* ≤ 0.05. When a significance level of *P* ≤ 0.01 was applied, the expressions of one or two transcripts of each of these ten published ginsenoside biosynthesis genes remained correlated with the variations of one or more of the mono- and total-ginsenoside contents (Supplementary Table S4). These results not only further verified the roles of these published genes in ginsenoside biosynthesis, but importantly, it also suggested that if a gene was significantly correlated in expression with the variations of the mono- and total-ginsenoside contents, it was highly likely, if not it was, involved in ginsenoside biosynthesis.

Therefore, we conducted the expression correlation analysis of all the 440 *PgCYP* transcripts with the variations of the nine mono- and total-ginsenoside contents in the four-year-old roots of the 42 cultivars. As a result, a total of 111 transcripts derived from 100 *PgCYP* genes were correlated in expression with the variations of the nine mono- and total-ginsenoside contents, when a significance level of *P* ≤ 0.05 was applied (Table 1; Supplementary Tables S5 and S6). For the nine mono-ginsenosides, the expressions of 9–46 transcripts per mono-ginsenoside were correlated with the variations of their contents in the four-year-old roots of 42 cultivars. For the total ginsenoside, the expressions of 48 transcripts were correlated with the variations of its content. When a significance level of *P* ≤ 0.01 was applied, a total of 62 transcripts derived from 59 *PgCYP* genes were correlated in expression with the variations of also all nine mono- and total-ginsenoside contents. For the nine mono-ginsenosides, the expressions of 3–18 transcripts per mono-ginsenoside were correlated with the variations of their contents. For the total ginsenoside, the expressions of 24 transcripts were correlated with the variations of its content. These results suggested that at least 100 gene members of the *PgCYP* gene superfamily are likely involved in ginsenoside biosynthesis in the four-year-old roots of the 42 cultivars at a confidence of >95%.

**Table 1:** The *PgCYP* genes that are significantly correlated with the variation of mono- and total-ginsenoside contents in the four-year-old roots of 42 cultivars.

Rg1	Re	Rf	Rb1	Rg2	Rc	Rb2	Rb3	Rd	TS
*PgCYP020*	*PgCYP056*	*PgCYP144*	*PgCYP001*	*PgCYP100*	*PgCYP126*	*PgCYP070*	*PgCYP090*	*PgCYP001*	*PgCYP001*
*PgCYP070*	*PgCYP096*	*PgCYP200*	*PgCYP014*	*PgCYP106*	*PgCYP149*	*PgCYP114*	*PgCYP138*	*PgCYP020*	*PgCYP014*
*PgCYP071*	*PgCYP128*	*PgCYP205*	*PgCYP020*	*PgCYP126*	*PgCYP234*	*PgCYP138*	*PgCYP168*	*PgCYP025*	*PgCYP020*
*PgCYP137*	*PgCYP138*	*PgCYP220*	*PgCYP056*	*PgCYP171*	*PgCYP247*	*PgCYP176*	*PgCYP176*	*PgCYP070*	*PgCYP056*
*PgCYP144*	*PgCYP176*	*PgCYP222*	*PgCYP070*	*PgCYP250*	*PgCYP338*	*PgCYP177*	*PgCYP186*	*PgCYP136*	*PgCYP070*
*PgCYP161*	*PgCYP177*	*PgCYP227*	*PgCYP096*	*PgCYP274-1*	*PgCYP347-2*	*PgCYP187*	*PgCYP187*	*PgCYP137*	*PgCYP136*
*PgCYP167*	*PgCYP187*	*PgCYP240*	*PgCYP136*	*PgCYP279*	*PgCYP347-4*	*PgCYP188*	*PgCYP188*	*PgCYP171*	*PgCYP137*
*PgCYP191*	*PgCYP188*	*PgCYP247*	*PgCYP137*	*PgCYP310-2*	*PgCYP359-2*	*PgCYP200*	*PgCYP218*	*PgCYP173*	*PgCYP173*
*PgCYP210*	*PgCYP196*	*PgCYP251*	*PgCYP173*	*PgCYP335-1*	*PgCYP376-3*	*PgCYP205*	*PgCYP237*	*PgCYP177*	*PgCYP176*
*PgCYP227*	*PgCYP198*	*PgCYP292*	*PgCYP177*		*PgCYP385*	*PgCYP218*	*PgCYP239*	*PgCYP188*	*PgCYP177*
*PgCYP301*	*PgCYP205*	*PgCYP339*	*PgCYP188*		*PgCYP396*	*PgCYP237*	*PgCYP278*	*PgCYP205*	*PgCYP188*
*PgCYP309*	*PgCYP218*	*PgCYP347-2*	*PgCYP205*			*PgCYP239*	*PgCYP302*	*PgCYP210*	*PgCYP205*
*PgCYP319*	*PgCYP237*	*PgCYP359-1*	*PgCYP211*			*PgCYP247*	*PgCYP303*	*PgCYP225*	*PgCYP218*
*PgCYP332*	*PgCYP239*	*PgCYP396*	*PgCYP218*			*PgCYP267*	*PgCYP315-1*	*PgCYP233*	*PgCYP225*
*PgCYP366*	*PgCYP247*		*PgCYP225*			*PgCYP278*	*PgCYP326*	*PgCYP234*	*PgCYP233*
*PgCYP374*	*PgCYP260*		*PgCYP233*			*PgCYP291*	*PgCYP348-1*	*PgCYP247*	*PgCYP234*
*PgCYP376-1*	*PgCYP267*		*PgCYP234*			*PgCYP292*	*PgCYP376-1*	*PgCYP258*	*PgCYP237*
*PgCYP376-4*	*PgCYP274-1*		*PgCYP247*			*PgCYP311*	*PgCYP376-3*	*PgCYP261-2*	*PgCYP239*
	*PgCYP278*		*PgCYP258*			*PgCYP313-2*	*PgCYP376-5*	*PgCYP263*	*PgCYP247*
	*PgCYP285-1*		*PgCYP261-2*			*PgCYP326*	*PgCYP378*	*PgCYP264*	*PgCYP258*
	*PgCYP292*		*PgCYP263*			*PgCYP335-2*		*PgCYP267*	*PgCYP261-2*
	*PgCYP311*		*PgCYP264*			*PgCYP348-1*		*PgCYP269*	*PgCYP263*
	*PgCYP313-1*		*PgCYP269*			*PgCYP376-1*		*PgCYP274-1*	*PgCYP264*
	*PgCYP313-2*		*PgCYP272*			*PgCYP376-5*		*PgCYP274-2*	*PgCYP267*
	*PgCYP326*		*PgCYP278*					*PgCYP282*	*PgCYP269*
	*PgCYP339*		*PgCYP280*					*PgCYP294*	*PgCYP274-1*
	*PgCYP348-1*		*PgCYP282*					*PgCYP300*	*PgCYP274-2*
	*PgCYP361*		*PgCYP285-1*					*PgCYP305*	*PgCYP278*
	*PgCYP369*		*PgCYP292*					*PgCYP309*	*PgCYP285-1*
	*PgCYP376-1*		*PgCYP294*					*PgCYP310-1*	*PgCYP292*
	*PgCYP376-5*		*PgCYP303*					*PgCYP311*	*PgCYP294*
			*PgCYP305*					*PgCYP336*	*PgCYP300*
			*PgCYP307*					*PgCYP348-2*	*PgCYP303*
			*PgCYP309*					*PgCYP363-1*	*PgCYP305*
			*PgCYP311*					*PgCYP369*	*PgCYP309*
			*PgCYP336*						*PgCYP310-1*
			*PgCYP339*						*PgCYP311*
			*PgCYP341*						*PgCYP313-2*
			*PgCYP342*						*PgCYP326*
			*PgCYP348-2*						*PgCYP339*
			*PgCYP361*						*PgCYP341*
			*PgCYP363-1*						*PgCYP342*
			*PgCYP363-2*						*PgCYP347-2*
			*PgCYP365*						*PgCYP348-1*
			*PgCYP369*						*PgCYP348-2*
			*PgCYP412*						*PgCYP361*
									*PgCYP363-1*
									*PgCYP369*
18	31	14	46	9	11	24	20	35	48

Mono-ginsenosides, Rg1, Re, Rf, Rb1, Rg2, Rc, Rb2, Rb3 and Rd; Total ginsenosides, TS. For detail, see Supplementary Table S5.

### Association study of the candidate *PgCYP* genes involved in ginsenoside biosynthesis

Furthermore, we carried out mutation analysis of these 100 *PgCYP* genes that were significantly correlated in expression with the variation of ginsenoside contents among the 42 cultivars. The single marker analysis method of QTL mapping^37^ and the candidate gene approach of genome-wide association study^38^ were used for the analysis. Because gene SNP/InDel mutation analysis has been widely used for gene functional analysis, this analysis would identify the genic or functional SNP/InDel markers for these 100 *PgCYP* genes and also provide an additional line of evidence on their functionality in ginsenoside biosynthesis. We identified a total of 724 SNPs/InDels from these 100 *PgCYP* genes. Association analysis showed that 78 of the 724 SNPs/InDels (10.8%) that were contained in 18 of the 100 *PgCYP* genes were associated with the variation of ginsenoside contents, when a significance level of *P* ≤ 0.05 was applied. These 18 *PgCYP* genes included all three *PgCYP* genes, *CYP716A47*, *CYP716A53v2* and *CYP716A52v2* previously cloned by Han *et al*.^17–19^. The reason that the remaining 646 SNPs were not associated with the variation of ginsenoside contents was because they were synonymous nucleotide substitutions that highly likely had no biological effects as they did not change the coding protein sequences^39^. Therefore, these 78 SNP/InDel mutations had significant influences on the contents of all nine mono- and total-ginsenosides and different mutations of a *PgCYP* gene might influence different ginsenosides. When a significance level of *P* ≤ 1.0E-04 (according to the Bonferroni multiple testing correction, *P* = 0.05/100 = 5.0E-04) was applied, the five SNPs and three InDels contained in six of the 18 *PgCYP* genes remained significantly associated with the variations of the ginsenosides (Table 2). Therefore, these six *PgCYP* genes were considered to be involved in the ginsenoside biosynthesis or to be the candidate genes involved in the ginsenoside biosynthesis. Of the eight SNP or InDel mutations, three (37.5%) were non-synonymous, one (12.5%) was synonymous, three (37.5%) were ORF-shifted, and one (12.5%) was not found in ORF. According to Graur and Li^39^, a vast majority of synonymous substitutions have no biological impacts, but recent studies showed that some of the synonymous SNPs or nucleotide substitutions may have biological functions^40^.

**Table 2:** The *PgCYP* genes that are significantly correlated with the variation of ginsenoside contents in the four-year-old roots of 42 farmers’ cultivars and also have mutations that are significantly associated with ginsenoside contents.

Gene	Position nucleotide mutation	Mutation type	Effect % (*P*-value)				
			Rg1	Re	Rc	Rb2	TS
*PgCYP149*	70_CT_C	ORF shift					
	73_C_CGTGTGA AAATAAAAGAA ACCCAGAACCCA TTAAAGCACCAG	ORF shift	52.0 (5.50E-04)				30.7 (9.38E-05)
*PgCYP218*	667_A_G	S				69.08 (2.90E-05)	
	681_T_C	NS				63.81 (3.60E-05)	
*PgCYP222*	647_T_G	NS	62.7 (1.63E-04)				
*PgCYP305*	1214_A_AAT	ORF shift			69.9 (1.19E-04)		
*PgCYP341*	140_G_A	Not in ORF		73.1 (8.69E-04)			
*PgCYP385*	468_G_A	NS			52.6 (8.08E-04)		

Mono-ginsenosides, Rg1, Re, Rc and Rb2; Total ginosenosides, TS; S, synonymous substitution; NS, non-synonymous substitution; ORF, open-reading frame.

### Characterization of the candidate *PgCYP* genes involved in ginsenoside biosynthesis

**Figure 1:**
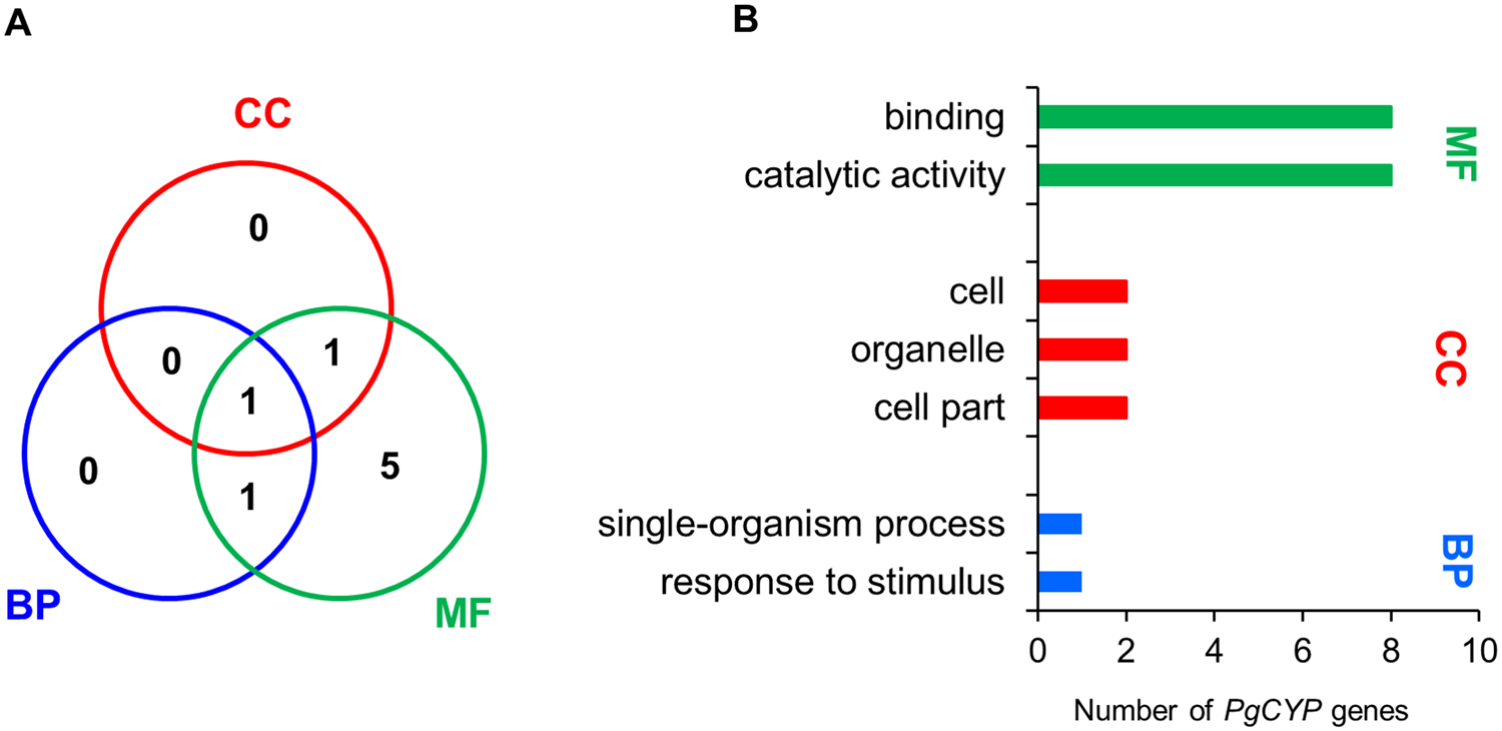
GO functional categorization of the six ginsenoside biosynthesis candidate *PgCYP* genes and the three published ginsenosides biosynthesis *PgCYP* genes (*CYP716A47*, *CYP716A53v2* and *CYP716A52v2*). (**A**) Venn diagram of eight of the *PgCYP* genes among the three primary functional categories: biological process (BP), molecular function (MF) and cellular component (CC). One of the *PgCYP* genes was not assigned to a GO term. (**B**) The eight *PgCYP* genes were categorized into seven functional subcategories (Level 2), including 2 MF subcategories, 3 CC subcategories and 2 BP subcategories.

Because the SNP or InDel mutations of six of the 100 *PgCYP* genes that were significantly correlated with the variations of ginsenoside contents were also associated with ginsenoside biosynthesis at *P* ≤ 1.0E-04 (Table 2), we further analyzed these six *PgCYP* genes, along with the three published *PgCYP* genes, *CYP716A47*, *CYP716A53v2* and *CYP716A52v2*^17–19^, to have an insight into how the genes of the *PgCYP* gene superfamily are involved in the process. First, we functionally *in silico* categorized the *PgCYP* genes by GO term and pathway mapping to the KEGG pathway database^41^. Of the nine *PgCYP* genes, eight were assigned to one or more GO terms and categorized into all three primary functional categories, biological process (BP), molecular function (MF) and cellular component (CC) (Fig. 1A). All of them had categorized functions in Binding and Catalytic Activity (Fig. 1B; Supplementary Table S7), suggesting their biochemical function. Nevertheless, none of these six *PgCYP* genes and the three published ginsenoside biosynthesis *PgCYP* genes was mapped to any of the metabolic pathways related to ginsenoside biosynthesis documented in the KEGG pathway database. This result suggested that additional research remains to further develop the pathway(s) responsible for ginsenoside biosynthesis.

**Figure 2:**
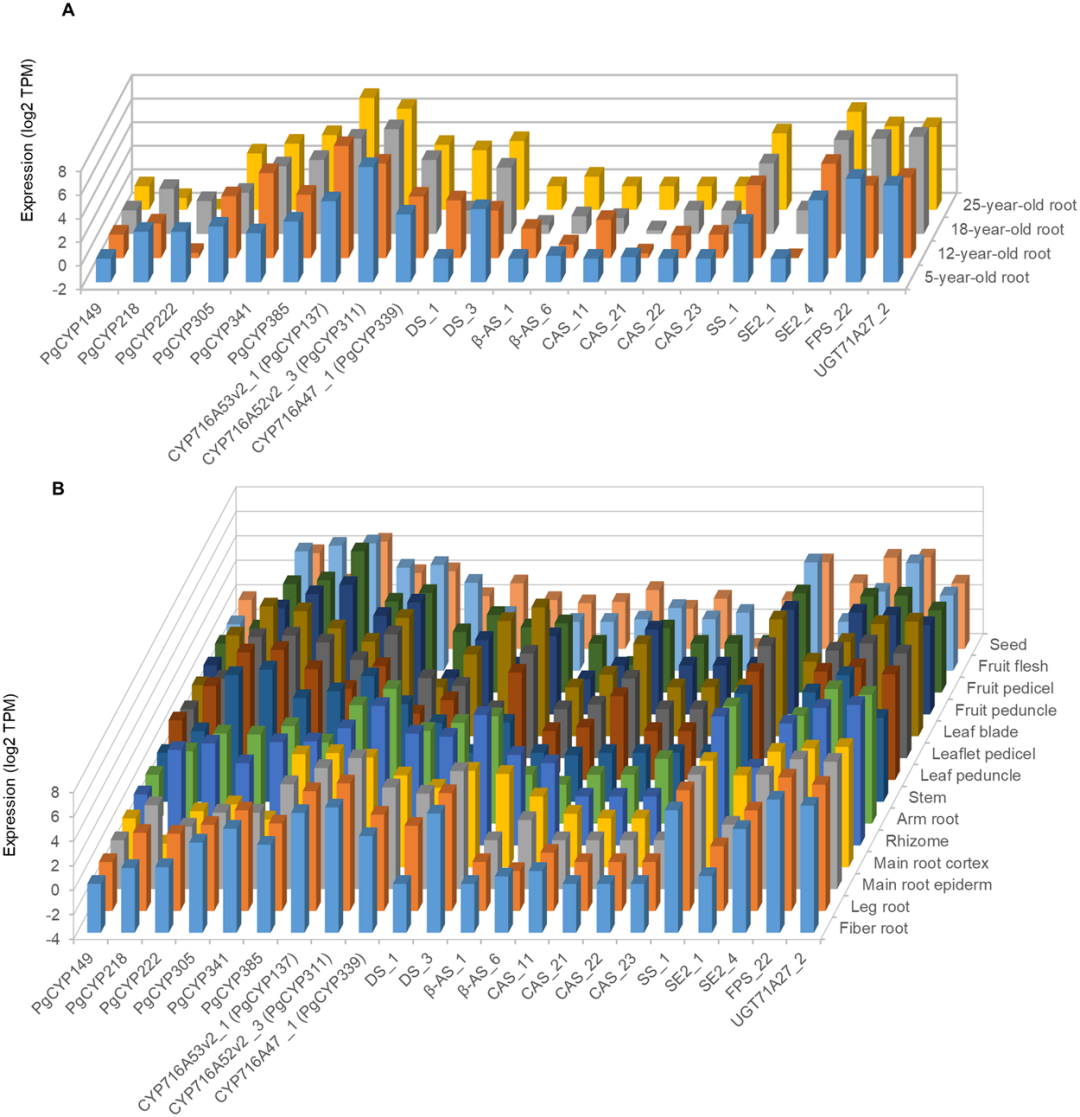
Temporal-spatial expressions of the six ginsenoside biosynthesis candidate *PgCYP* genes and the previously cloned ginsenosides biosynthesis genes. *CYP716A53v2_1*, *CYP716A52v2_3*, *CYP716A47_1*, *DS_1*, *DS_3*, *β-AS_1*, *β-AS_6*, *CAS_11*, *CAS_21*, *CAS_22*, *CAS_23 SS_1*, *SE2_1*, *SE2_4*, *FPS_22* and *UGT71A27_2*, are previously cloned ginsenosides biosynthesis genes, while the six ginsenoside biosynthesis candidate *PgCYP* genes were identified in this study. The suffix of each gene, such as “_1” and “_3” to *DS*, indicates the transcript of the gene. (**A**) Expressions of the genes in the roots of differently-aged plants. (**B**) Expressions of the genes in different tissues of a four-year-old plant.

Next, we examined the expression activities of the six *PgCYP* genes identified in this study and those 10 published ginsenoside biosynthesis genes, including three *PgCYP* genes and seven genes previously cloned from other gene families, at different developmental stages and in different tissues. In the roots of differently-aged plants, from 5 to 25 years old, all 16 of the genes but *PgCYP149* expressed at a developmental stage, with a variation from < 1.0 TPM to 220 TPM (Fig. 2A; Supplementary Table S8). In the different tissues of a four-year-old plant, all of the 16 analyzed genes expressed in four or more tissues, varying from < 1.0 TPM to 180 TPM, while for a same gene, its expression also varied dramatically across tissues (Fig. 2B; Supplementary Table S8).

**Figure 3:**
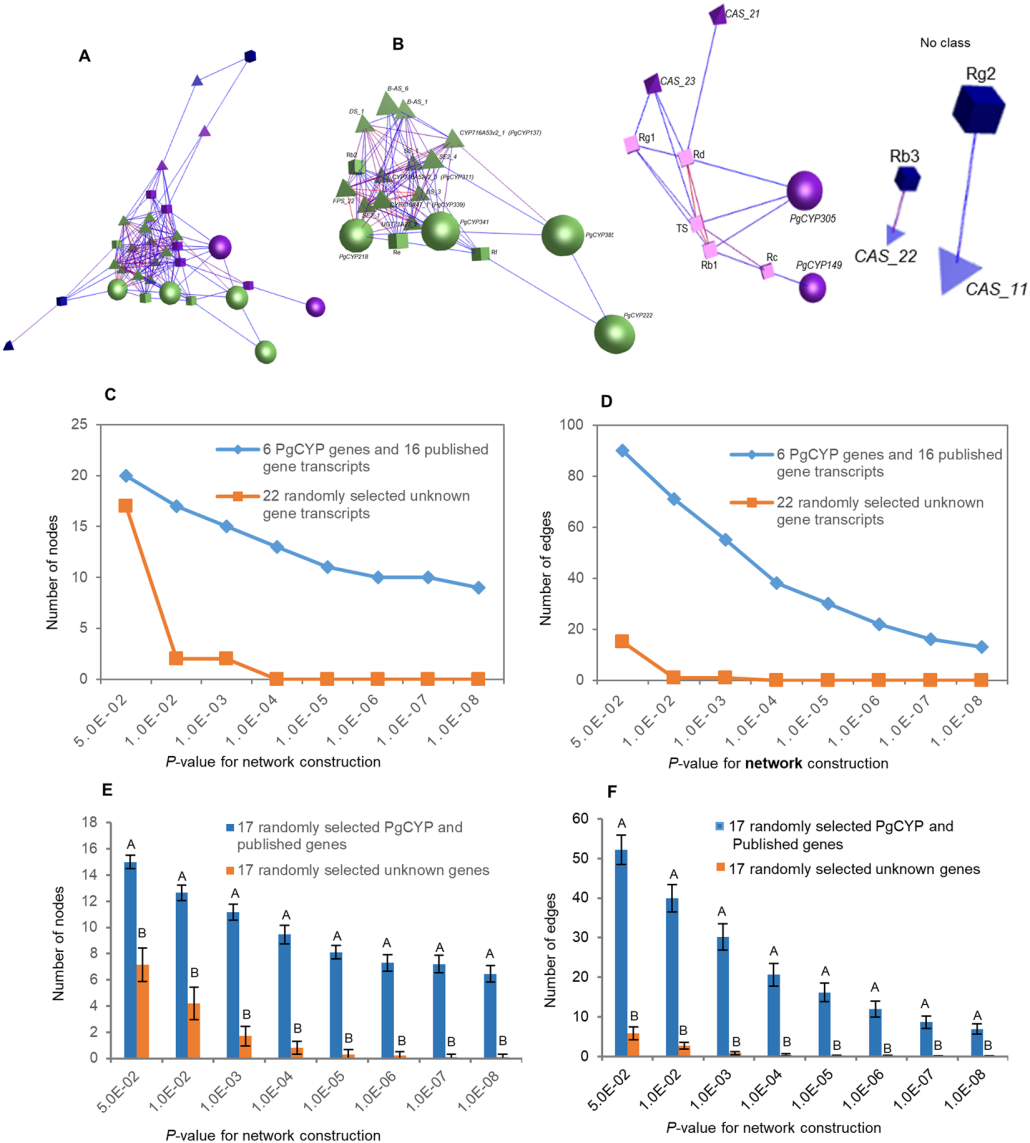
Network analysis of the six ginsenoside biosynthesis candidate *PgCYP* genes and all 10 published ginsenoside biosynthesis genes. The network was constructed based on their expressions in the four-year-old roots of 42 cultivars. (**A**) The co-expression network constructed from the six *PgCYP* genes identified in this study indicated by balls, 16 transcripts of the 10 published ginsenoside biosynthesis genes indicated by diamonds, and 9 mono- and total ginsenosides indicated by cubes. The network was constructed at *P* ≤ 5.0E-02. (**B**) The clusters constituting the network. Different clusters are indicated by different colors. (**C**) Tendency that these ginsenoside biosynthesis related genes form a network, with the randomly-selected ginseng unknown genes as controls: variation in number of nodes. (**D**) Tendency that these ginsenoside biosynthesis related genes form a network, with the randomly-selected ginseng unknown genes as controls: variation in number of edges. (**E**) Statistics of variation in number of nodes in the network. (**F**) Statistics of variation in number of edges in the network. Different capital letters, significant at *P* ≤ 0.01. Error bar, standard deviation for 20 replications.

Third, we studied whether these genes, including those *PgCYP* genes identified in this study and those previously published, were related in functionality in ginsenoside biosynthesis. This study would also provide an additional line of evidence on whether these *PgCYP* genes are involved in ginsenoside biosynthesis. This is because the genes involved in a biological process or controlling a phenotype significantly tend to form a single co-expression network (MPZ, Y.-H. Liu and H.-B Zhang, personnel communication). Therefore, we constructed the co-expression network of these genes in the four-year-old roots of the 42 cultivars with the nine mono- and total-ginsenoside contents (Fig. 3A). The network consisted of all the six newly identified ginsenoside biosynthesis candidate *PgCYP* genes, all 10 published ginsenoside biosynthesis genes (16 transcripts) and all nine mono-ginsenosides and total ginsenoside, 184 co-expression edges and 2 clusters (Fig. 3B). The ginsenoside biosynthesis candidate *PgCYP* genes identified in this study closely co-expressed with the published ginsenoside biosynthesis genes and the mono- and total-ginsenoside contents in both the entire network and its individual clusters. The network formation of these genes was far more tended than those randomly-selected unknown ginseng genes (Fig. 3C–F), thus further verifying that these *PgCYP* genes were involved in ginsenoside biosynthesis and suggesting that the *PgCYP* genes are correlated in functionality in the process.

**Figure 4:**
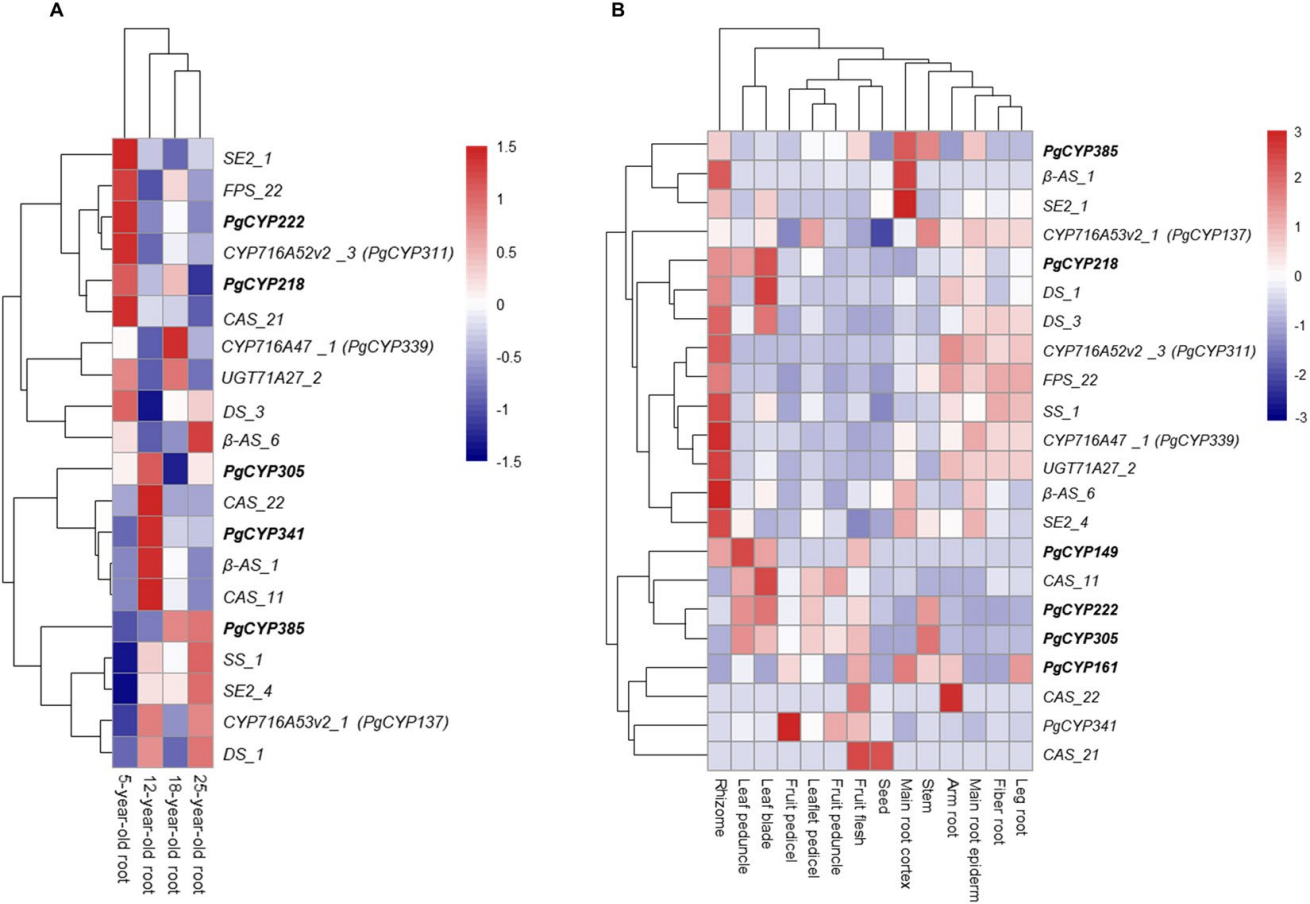
Expression heatmap of the six ginsenoside biosynthesis candidate *PgCYP* genes in different aged roots and tissues. The six ginsenoside biosynthesis candidate *PgCYP* genes newly identified in this study are bolded. All published genes involved in ginsenosides biosynthesis, *CYP*, *AS*, *CAS*, *SE*, *SS*, *UGT*, *FPS* and *DS*, were used as controls. (**A**) Heatmap of the genes expressed in the roots of differently-aged plants. One of the six ginsenoside biosynthesis candidate *PgCYP* genes did not express in the roots. (**B**) Heatmap of the genes expressed in the tissues of a four-year-old plant.

Finally, we investigated whether these genes were commonly regulated, with an attempt to identify expression signatures that are associated with a particular developmental stage or tissue through expression heatmap analysis. The result showed that common regulations existed at different developmental stages of roots for some of the genes (Fig. 4A), but were not identified in different tissues of a four-year-old plant (Fig. 4B). For instance, it appeared that *β-AS_1* and *CAS_11* were commonly regulated in the roots of ginseng aged from 5 years old through 25 years old (Fig. 4A). However, the expression patterns of these genes were well distinguished among the roots of differently aged plants (Fig. 4A) and the different tissues of a four-year-old plant (Fig. 4B). The unique expression patterns of these genes in differently-aged roots and different tissues, therefore, provide useful signatures for ginseng research and applications in ginsenoside biosynthesis and production.

## Discussion

The *CYP* genes encoding cytochrome P450 play important roles in numerous biological processes in plants and other organisms. Han *et al*.^17–19^ identified three *CYP* genes, *CYP716A53v2*, *CYP716A52v2* and *CYP716A47*, from *P*. *ginseng* and showed that they are involved in ginsenoside biosynthesis. This study has identified 100 *PgCYP* genes including these three published *CYP* genes whose transcript expressions are significantly correlated with the variations of one or more nine mono- and/or total-ginsenoside contents in 42 ginseng cultivars. Given that the functions of all three published ginsenoside biosynthesis *CYP* genes, *CYP716A53v2*, *CYP716A52v2* and *CYP716A47*^17–19^, were determined by gene expression analysis with RNAi followed by association of the repressed gene expression with the ginsenoside contents, the gene transcript expression – ginsenoside content correlation result provides a strong line of evidence on the contributions of these 97 new *PgCYP* genes identified in this study to ginsenoside biosynthesis. Furthermore, of these 97 *PgCYP* genes newly identified in this study, six were also identified to contain SNPs and/or InDels that significantly increased or decreased the content(s) of one or more mono- and/or total-ginsenosides in the four-year-old roots of ginseng cultivars (*P* ≤ 1.0E-04). Moreover, the six *PgCYP* genes newly identified in this study formed a single strong co-expression network with all the published ginsenoside biosynthesis genes. If the gene expression-ginsenoside content correlation, gene mutation-ginsenoside content variation association and newly identified *PgCYP* gene-published ginsenoside biosynthesis genes co-expression network are taken into account as three of the criteria for the genes involved in ginsenoside biosynthesis, this study has identified at least 6 new *PgCYP* genes involved in ginsenoside biosynthesis. Therefore, these six *PgCYP* genes have provided gene resources important for ultimate identification of the *PgCYP* gene involved in ginsenoside biosynthesis and the *PgCYP* genic SNPs/InDels have provided useful DNA markers for enhanced ginseng research and breeding.

Systems analysis of these six newly identified ginsenoside biosynthesis candidate *PgCYP* genes, along with the ten ginsenoside biosynthesis genes previously cloned from the *PgCYP* and other gene families, and the ginsenoside contents, provides a first genome-wide insight into how the *PgCY*P gene superfamily is involved in ginsenoside biosynthesis. First, although the six *PgCYP* genes all belong to the *PgCYP* gene superfamily and all are likely involved in the process of ginsenoside biosynthesis, they were categorized into seven GO subcategories at Level 2. Therefore, the *PgCYP* genes are likely involved in multiple biochemical reactions of ginsenoside biosynthesis, which is in consistence with the fact that multiple mono-ginsenosides, including the nine mono-ginsenosides investigated in this study have been found from ginseng. Second, the six *PgCYP* genes dramatically differentially express across tissues and developmental stages, suggesting that different members of the *PgCYP* genes may be responsible for ginsenoside biosynthesis individually or in group in different tissues and at different developmental stages. Third, some of the ginsenoside biosynthesis genes show different levels of common regulations across developmental stages, thus providing unique signatures for each developmental stage. Finally, the six ginsenoside biosynthesis candidate *PgCYP* genes and three published ginsenoside biosynthesis *PgCYP* genes clearly function collaboratively with each other and with those ginsenoside biosynthesis genes from other gene families in the process of ginsenoside biosynthesis, which is demonstrated by their co-expression in their network. Further, the co-expression network of these ginsenoside biosynthesis candidate genes with the published ginsenoside biosynthesis genes also suggests that the ginsenoside biosynthesis process consists of many pathways of a single network, while the different *PgCYP* genes are involved in different pathways of the *PgCYP* gene network. This may at least partly explain why this study failed to map the six ginsenoside biosynthesis candidate PgCYP genes and the three published ginsenoside biosynthesis genes^17–19^ to any of the KEGG metabolic pathways related to ginsenoside biosynthesis. Nevertheless, the network of the *PgCYP* genes provides information necessary for advanced research and applications of ginsenoside biosynthesis, such as regulation of ginsenoside biosynthesis by gene editing or RNAi and engineering of ginsenoside biosynthesis by genetic transformation.

## Conclusions

Previous studies identified three *PgCYP* genes that are involved in the ginsenoside biosynthesis^17–19^. This study has identified at least six additional *PgCYP* genes that are highly likely involved in the ginsenoside biosynthesis. If all the *PgCYP* genes whose expressions were significantly correlated with the variations of nine mono- and total-ginsenoside contents in different ginseng cultivars are considered to be candidates for ginsenoside biosynthesis, the number of newly identified ginsenoside biosynthesis candidate *PgCYP* genes should be 97. Moreover, the six *PgCYP* genes form a single network with the three published *PgCYP* genes and seven published other genes involved in ginsenoside biosynthesis. These results, therefore, strongly suggest that these six new *PgCYP* genes are also involved in the ginsenoside biosynthesis and have provided a set of *PgCYP* genic SNP/InDel markers for advanced ginsenoside biosynthesis research and breeding in ginseng and related species.

## Materials and Methods

### Ginseng transcriptome databases and *PgCYP* genes

Three transcriptome databases of Jilin ginseng, including transcript sequences and expressions, were used for this study. The first one was developed from 14 tissues of a four-year-old ginseng plant^42^, the second from the roots of 5-, 12-, 18- and 25-year-old ginseng plants^42^ and the third from the four-year-old roots of 42 cultivars or genotypes representing the genetic variations of ginseng in its major origin and production center, Jilin, China^36^. These plant materials were all sampled from the major origin and production regions of ginseng in Jilin, China (Supplementary Table S3). These three transcriptome data were all sequenced using the Illumina platform Hiseq 2000 with a single flow cell, a module of 100 PE (100-nucleotide paired ends), and a sequencing depth of approximately 14 million clean reads per sample.

We previously identified 440 transcripts that were derived from 414 *PgCYP* genes^35^. These *PgCYP* genes were defined with numbers of 001 through 414. The different transcripts derived from a single *PgCYP* gene were defined by suffixing the name of the *PgCYP* gene with “−1”, “−2”, etc., such as *PgCYP274-1* and *PgCYP274-2*.

### Expressions of *PgCYP* transcripts

Since gene expression is essential for gene function and gene function determination, and different transcripts alternatively spliced from a single gene may have different biological functions^34^, we quantified the expression activity of each *PgCYP* transcript in different tissues, at different developmental stages and in different genotypes or cultivars. This was accomplished by extracting the expression profile and activity of each *PgCYP* transcript from the three transcriptome databases described above. The expression of each transcript was determined using the RSEM software^43^ bundled with the Trinity software (for detail, see ref.^42^). Zhang *et al*.^44^ showed that the expressions of individual transcripts quantified by shotgun RNA-seq, as measured in this study, were high reproducible between biological replicates.

### Ginsenoside content assay

The contents of nine mono-ginsenosides, Rg1, Re, Rf, Rg2, Rb1, Rc, Rb2, Rb3 and Rd, were assayed by HPLC as described by Zhang *et al*.^2^. The standards for Rg1, Re, Rf, Rg2, Rb1, Rc, Rb2, Rb3 and Rd were purchased from the National Institute for the Control of Pharmaceutical and Biological Products (Beijing, China). The four-year-old roots of 42 cultivars were sampled from a field trial located in Jingyu, Jilin, China and used as plant materials. The tissues used for this experiment were parts of the root tissues that were previously used to quantify the expressions of *PgCYP* transcripts described above^36^. The HPLC experiment was performed using LC-2010A HPLC equipped with LC-2010A liquid chromatography pump, LC-2010A autosampler, SHIMADZU C18 (4.6 mm × 250 mm, 5 μm) column, and CLASS-VP chromatographic workstation. We obtained the proper phenotype data for all nine mono-ginsenosides in all 42 cultivars analyzed in this study. The total ginsenoside content was the sum of the nine mono-ginsenosides.

### Identification of *PgCYP* genes significantly correlated with ginsenoside contents

According to the central dogma of biology that the phenotype of a trait, including the ginsenoside contents in a tissue, is a result of gene expression activity, we hypothesized that the variation of ginsenoside contents in a tissue of a population must be related with the expression activity of the genes involved in ginsenoside biosynthesis. Therefore, to genome-wide identify the candidate genes that are involved in ginsenoside biosynthesis, we conducted correlation analysis between the expressions of the *PgCYP* gene transcripts and the variations of nine mono- and total-ginsenoside contents in the four-year-old roots of 42 cultivars using the statistical package IBM SPSS Statistics 22. The two-tailed significance levels were employed for the analysis.

### Gene mutation analysis

All *PgCYP* genes that were significantly correlated in expression with the variations of the ginsenoside contents were subjected to analysis for genic or functional SNPs (single nucleotide polymorphisms) and InDels (nucleotide insertions and deletions) in the 42 cultivars using SAMtools^45,46^. The RNA-seq clean reads of each cultivar^36^ were used for the analysis, with the sequences of the genes expressed in 14 tissues of a four-year-old plant^42^ as the reference. The SNPs and InDels present only in four or more of the cultivars were used for mutation analysis. This was because the *PgCYP* transcripts had an average length of 1,306 bp (see below), the probability that four cultivars have a same SNP or InDel by chance, such as resulted from sequencing and/or transcript assembly errors, would be *P* = 3.4E-13 (1/1306*1/1306*1/1306*1/1306). This approach would allow almost all, if not all, of the SNPs or InDels that resulted from sequencing or transcript assembly errors to be excluded from the analysis. The SNPs or InDels were subjected to association analysis with the variations of the ginsenoside contents according to the single marker analysis method of QTL mapping^37^ and the candidate gene approach of genome-wide association study^38^. The SNPs and InDels that were associated with the variations of ginsenoside contents at a two-tailed significance level of *P* ≤ 1.0e-04 were further analyzed in open reading frame (ORF) using the ORF Finder available at NCBI.

### Annotation and functional differentiation estimation of the candidate *PgCYP* genes involved in the ginsenoside biosynthesis

Gene ontology (GO) analysis is a widely used sequence identity-based bioinformatic tool to annotate and *in silico* functionally categorize genes. Therefore, to further characterize the candidate *PgCYP* genes for ginsenoside biosynthesis we annotated them and estimated their functional differentiation by GO analysis with the Blast2GO software^47^.

### Co-expression network and expression heatmap analysis

The co-expression network of the ginseonside biosynthesis candidate *PgCYP* genes newly identified in this study, the published ginsenoside biosynthesis *PgCYP* genes, the published ginsenoside biosynthesis genes previously cloned from other gene families, and the nine mono- and total-ginsenoside contents, and the expression heatmaps of the *PgCYP* genes and the published ginsenoside biosynthesis genes were constructed as previously described^42^.

## Electronic supplementary material

Table S1

Table S2

Table S3

Table S4

Table S5

Table S6

Table S7

Table S8
